# Supplementary material for: Genome-Wide Analysis and Identification of the Aux/IAA Gene Family in Peach
Source: Int J Mol Sci. 2019 Sep 23;20(19):4703. doi: 10.3390/ijms20194703 (PMC6801721; doi:10.3390/ijms20194703)
Supplement: Supplementary file 1 [file ijms-20-04703-s001.pdf]

## Supplementary Material

**Table S1.** Primers used for PCR of *PpIAA* ORFs

| Gene ID | Transcript name | Forward primer (5′-3′) | Reverse primer (5′-3′) |
|---|---|---|---|
| *PpIAA1* | *ppa011843m* | ATGGAAGGGTCAGTGAAATATGAGA | TCTCATATTTCACTGACCCTTCCAT |
| *PpIAA5* | *ppa011935m* | ATGGCCAAAGAAGGTTTAG | TTATTTAGGATCATCTTTCATAGTT |
| *PpIAA9* | *ppa006744m* | ATGTCACCACCGCTGCTTGGTG | GTTCCTGTTCCTGCACTTCTCCATG |
| *PpIAA11* | *ppa008953m* | ATGGAGGGTGTTTTGGGTAGTG | TATCGGCTGGCATCTTTGC |
| *PpIAA13* | *ppa010871m* | ATGGAGCTTCAACTGGGTC | ATCACTCTTCTTAACCAATTTG |
| *PpIAA14* | *ppa010342m* | ATGGGGTTTGAAGAGACGGAG | GCTTCTGTTCTTGCATTTCTCC |

**Table S2.** Primers used for qRT-PCR of *PpIAA* gene family members

| Gene ID | Transcript name | Forward primer (5′-3′) | Reverse primer (5′-3′) |
|---|---|---|---|
| *PpIAA1* | *ppa011843m* | GATCACACAGATGCCCCTCC | GCTCCATCGACGCTTACTTTCA |
| *PpIAA2* | *ppa018535m* | CAAAGGCTACCCAGAACTCCTAA | CATCTCCAACCAGCATCCAGTCA |
| *PpIAA3* | *ppa011755m* | AGCTGCCAAAAGATGTGACCAA | CACATAAATCCCACAACCCTCC |
| *PpIAA5* | *ppa011935m* | AAAGCGAAACGAAGAACCAAG | GCTCCATCCATGCTAACCTTGAC |
| *PpIAA7* | *ppa010698m* | GCCAAGATGTTCAGTTCCTTCACC | CATCCAATCGCCATCCTTATCCTC |
| *PpIAA8* | *ppa007194m* | CACTGTTTCCTTTGCTTCCGTC | ACTTTTCACCTCCGAGTATCCA |
| *PpIAA9* | *ppa006744m* | GTATGCCACAATAGCTCGGAA | GAAGCCCAAGTCTAAGGTCTGT |
| *PpIAA11* | *ppa008953m* | TATAATGGCAGGAGCAACTAGAC | CACAGTACCAAGAAACATCCC |
| *PpIAA12* | *ppa009545m* | TGCTTCTTCTGCTGTTGCTGT | CCCGTCTTCCTTGTCATCTTC |
| *PpIAA13* | *ppa010871m* | CTTCAACTGGGTCTCGCTCT | TGGTCAAAGCTGCGTTTCTTGT |
| *PpIAA14* | *ppa010342m* | CTCTCTGATGCCCTAGCCAAAA | CCAACCAGCATCCAATCACC |
| *PpIAA15* | *ppa010303m* | AGCCTTAGACCAAATGTTCCC | TCCAGTCCCCATCCTTGTCTTC |
| *PpIAA16* | *ppa009254m* | GCTCCTTCACCATCGGTAACT | CCCACAAGCATCCAATCTCC |
| *PpIAA17* | *ppa011570m* | TTCTCCTTCTTGACCATCCGTAAT | TCCTACAAGCATCCAATCCCCA |
| *PpIAA18* | *ppa007663m* | GTGACAGCTATGAAAACCTCTCC | ACATCCCCAACAAGCATCCT |
| *PpIAA20* | *ppa011821m* | CTGGCTCATCATCTTCTTCCTCTC | GCCAGTTTATCAGTGCTTCCTCC |
| *PpIAA26* | *ppa013361m* | GAAACTCTCCCTTGCCATAGAT | GAGCAGAGTATATTCCCCATTGCC |
| *PpIAA27* | *ppa007893m* | TCTTCCTCTGCCCTCTCCACA | AGAGACCCCAATTCCAAGTCC |
| *PpIAA27′* | *ppa009134m* | AGGCACAGGTTGTAGGAT | TTCAGCACCATTTAGGAG |
| *PpIAA29* | *ppa010683m* | ATTGGGTCTTTCTCTGGCTCTTC | TGTGCTTTCCAAACCCAAACC |
| *PpIAA30* | *ppa020369m* | ACAACGCCATCATCATCTTCCC | AATCCGACGATGACCCCCT |
| *PpIAA32* | *ppa023002m* | GCAGCCTGAAACTTACCATCC | CTTTGAACTCCCTCTGCCTCT |
| *PpIAA33* | *ppa018956m* | GGACAAGACTCCCTGAAA | GCTACCATCCACGAACAT |
| *PpTEF-2* | *ppa001368m* | GTTGCCTTGGTCGGTCTTGA | ATTGAACAGCAACACGCACAA |

**Table S3** Analysis of amino acid sequence homology of PpIAA family members in peach.

| | **PpIA A1** | **PpIA A2** | **PpIA A3** | **PpIA A5** | **PpIA A7** | **PpIA A8** | **PpIA A9** | **PpIA A11** | **PpIA A12** | **PpIA A13** | **PpIA A14** | **PpIA A15** | **PpIA A16** | **PpIA A17** | **PpIA A18** | **PpIA A20** | **PpIA A26** | **PpIA A27** | **PpIA A27′** | **PpIA A29** | **PpIA A30** | **PpIA A32** | **PpIA A33** |
|---|---|---|---|---|---|---|---|---|---|---|---|---|---|---|---|---|---|---|---|---|---|---|---|
| PpIAA1 | - | | | | | | | | | | | | | | | | | | | | | | |
| PpIAA2 | 0.353 | - | | | | | | | | | | | | | | | | | | | | | |
| PpIAA3 | 0.429 | 0.392 | - | | | | | | | | | | | | | | | | | | | | |
| PpIAA5 | 0.600 | 0.590 | 0.626 | - | | | | | | | | | | | | | | | | | | | |
| PpIAA7 | 0.503 | 0.514 | 0.573 | 0.610 | - | | | | | | | | | | | | | | | | | | |
| PpIAA8 | 0.595 | 0.560 | 0.642 | 0.644 | 0.589 | - | | | | | | | | | | | | | | | | | |
| PpIAA9 | 0.575 | 0.598 | 0.658 | 0.623 | 0.593 | 0.377 | - | | | | | | | | | | | | | | | | |
| PpIAA11 | 0.728 | 0.720 | 0.752 | 0.768 | 0.746 | 0.769 | 0.765 | - | | | | | | | | | | | | | | | |
| PpIAA12 | 0.726 | 0.725 | 0.739 | 0.721 | 0.733 | 0.768 | 0.778 | 0.555 | - | | | | | | | | | | | | | | |
| PpIAA13 | 0.785 | 0.784 | 0.792 | 0.789 | 0.777 | 0.808 | 0.816 | 0.792 | 0.785 | - | | | | | | | | | | | | | |
| PpIAA14 | 0.547 | 0.545 | 0.615 | 0.653 | 0.359 | 0.545 | 0.571 | 0.745 | 0.753 | 0.788 | - | | | | | | | | | | | | |
| PpIAA15 | 0.643 | 0.634 | 0.673 | 0.659 | 0.603 | 0.676 | 0.671 | 0.784 | 0.775 | 0.806 | 0.590 | - | | | | | | | | | | | |
| PpIAA16 | 0.519 | 0.558 | 0.602 | 0.630 | 0.437 | 0.653 | 0.660 | 0.791 | 0.758 | 0.788 | 0.390 | 0.623 | - | | | | | | | | | | |
| PpIAA17 | 0.559 | 0.596 | 0.583 | 0.613 | 0.503 | 0.569 | 0.555 | 0.716 | 0.688 | 0.763 | 0.550 | 0.581 | 0.521 | - | | | | | | | | | |
| PpIAA18 | 0.750 | 0.733 | 0.782 | 0.768 | 0.790 | 0.806 | 0.827 | 0.795 | 0.791 | 0.806 | 0.776 | 0.795 | 0.805 | 0.750 | - | | | | | | | | |
| PpIAA20 | 0.774 | 0.747 | 0.763 | 0.756 | 0.779 | 0.777 | 0.750 | 0.679 | 0.722 | 0.749 | 0.781 | 0.749 | 0.790 | 0.744 | 0.772 | - | | | | | | | |
| PpIAA26 | 0.653 | 0.672 | 0.672 | 0.702 | 0.693 | 0.730 | 0.709 | 0.690 | 0.709 | 0.714 | 0.699 | 0.730 | 0.695 | 0.682 | 0.481 | 0.680 | - | | | | | | |
| PpIAA27 | 0.569 | 0.555 | 0.643 | 0.667 | 0.633 | 0.564 | 0.564 | 0.756 | 0.797 | 0.822 | 0.598 | 0.676 | 0.684 | 0.594 | 0.809 | 0.770 | 0.739 | - | | | | | |
| PpIAA27′ | 0.559 | 0.556 | 0.652 | 0.664 | 0.625 | 0.525 | 0.549 | 0.770 | 0.792 | 0.812 | 0.589 | 0.700 | 0.674 | 0.606 | 0.795 | 0.773 | 0.728 | 0.287 | - | | | | |
| PpIAA29 | 0.775 | 0.777 | 0.833 | 0.785 | 0.781 | 0.763 | 0.750 | 0.803 | 0.787 | 0.564 | 0.779 | 0.801 | 0.781 | 0.751 | 0.799 | 0.757 | 0.744 | 0.802 | 0.734 | - | | | |
| PpIAA30 | 0.733 | 0.744 | 0.712 | 0.755 | 0.762 | 0.751 | 0.780 | 0.741 | 0.730 | 0.787 | 0.770 | 0.763 | 0.785 | 0.750 | 0.792 | 0.587 | 0.736 | 0.765 | 0.736 | 0.799 | - | | |
| PpIAA32 | 0.739 | 0.756 | 0.778 | 0.765 | 0.757 | 0.777 | 0.751 | 0.711 | 0.704 | 0.777 | 0.777 | 0.779 | 0.758 | 0.758 | 0.794 | 0.791 | 0.752 | 0.749 | 0.744 | 0.765 | 0.783 | - | |
| PpIAA33 | 0.774 | 0.807 | 0.792 | 0.783 | 0.775 | 0.824 | 0.804 | 0.790 | 0.780 | 0.770 | 0.781 | 0.768 | 0.767 | 0.824 | 0.779 | 0.775 | 0.804 | 0.819 | 0.771 | 0.792 | 0.784 | 0.827 | - |

* Calculation model: p-distance.

The table shows the percent homology of the amino acid sequences of members of the PpIAA family in peach. Members of this family generally display 50%-80% sequence conservation in peach. The maximum similarity observed was between PpIAA3 and PpIAA29(83.3%), and the minimum similarity observed was between PpIAA27 and PpIAA27′(28%)
